# Understanding Cancer Clinical Trial Participation in Rural Communities: A Qualitative Focus Group Study

**DOI:** 10.1002/pon.70572

**Published:** 2026-08-09

**Authors:** Yetunde Tagurum, Jennifer N. Wells, Vickie Cater, Gigi A. Gonzales, Kimberly J. Kiplagat, Heidy Pickles, Sandra Baer, Kelli Mills, Crystal Clark, Stanley Jean‐Charles, Lindsey Murkerson, Christina White, Lynn Bentz, Kim Vigal, Gustavo Fonseca, Chandni Patel, Bradley J. Monk

**Affiliations:** ^1^ Florida Cancer Specialists & Research Institute West Palm Beach Florida USA; ^2^ Florida Cancer Specialists & Research Institute Fleming Island Florida USA; ^3^ Florida Cancer Specialists & Research Institute Tallahassee Florida USA; ^4^ Florida Cancer Specialists & Research Institute Port Charlotte Florida USA; ^5^ Florida Cancer Specialists & Research Institute Ocala Florida USA; ^6^ Florida Cancer Specialists & Research Institute Gainesville Florida USA; ^7^ Florida Cancer Specialists & Research Institute Lady Lake Florida USA; ^8^ Florida Cancer Specialists & Research Institute Lecanto Florida USA; ^9^ Florida Cancer Specialists & Research Institute Trinity Florida USA; ^10^ Florida Cancer Specialists & Research Institute Fort Myers Florida USA

**Keywords:** cancer clinical trials, clinical trial participation, focus groups, health behavior, patient navigation, rural health

## Abstract

**Background:**

Participation in cancer clinical trials is essential for advancing oncologic care, yet enrollment among rural populations remains disproportionately low. These communities face many hurdles including geographical distance from medical centers, healthcare workforce shortages, and deep‐seated medical mistrust that may contribute to survival inequities.

**Aims:**

To address these gaps, a Research Navigator program was launched through the Florida Cancer Specialists & Research Institute to engage rural Florida residents in focus group sessions to identify barriers to trial participation.

**Methods:**

Utilizing the Integrated Behavioral and Socioecological Framework (IBSF), the study conducted five semi‐structured focus groups with 30 participants recruited from rural Florida counties. Participants included cancer survivors, advocates, caregivers, and those currently in treatment. Discussions centered on knowledge, experiences, perceived barriers, and willingness to participate in cancer clinical trials.

**Results:**

Although most participants expressed awareness of cancer clinical trials, focus group discussions revealed misconceptions regarding trial purpose, process, and accessibility. Across groups, commonly reported barriers to participation included limited access to reliable transportation, financial concerns, and mistrust of medical professionals and healthcare systems. Thematic analysis of focus group transcripts yielded four themes: (A) perception and lived experience, (B) trial information dissemination, (C) enrollment impediments, and (D) intervention strategies and implementation.

**Conclusion:**

In rural settings, decisions regarding cancer clinical trial participation are shaped by a complex interplay of trust, logistics, and information access. This study emphasizes the importance of viewing trials as routine care and suggests that navigator‐led outreach and patient‐centric technology are essential for creating equitable access.

## 1 Background

### 1.1 Introduction and Literature Review

Cancer clinical trials (CCTs) are essential for advancing care, improving survival outcomes, and developing innovative therapeutic approaches. Despite their importance, participation in clinical trials remains consistently low, with substantial disparities affecting patients residing in rural communities. Estimates indicate that 5%–8% of adult cancer patients enroll in clinical trials, with participation rates declining further among individuals living in geographically underserved areas [1, 2]. Rural populations experience persistent structural and geographical barriers that limit access to specialized oncology services and research opportunities, contributing to inequities in cancer outcomes and survival [3, 4]. Geographic disparities in healthcare infrastructure, including shortages of oncology specialists and limited availability of clinical research programs, further exacerbates these challenges [5].

The underrepresentation of rural populations in CCTs is driven by multiple interrelated factors. Distance from medical centers, transportation barriers, financial constraints, and limited awareness of available research opportunities are consistently identified as significant obstacles [6, 7]. According to The American Association for Cancer Research, misconception about clinical research and safety concerns may reduce willingness to participate among the underserved populations [8]. Healthcare workforce shortages in rural communities further restrict opportunities for patients to receive information, referrals or support related to clinical trial enrollments [5]. Addressing these disparities requires targeted multilevel interventions that improve access to clinical trials, enhance patient education, and strengthen connection between rural health care systems and research institutions [9].

The purpose of this manuscript is to elucidate the barriers that rural Floridians face when considering clinical trial participation. Furthermore, we aim to understand strategies to improve access to clinical trials through targeted interventions. The study highlights the structural, logistical, and emotional barriers to clinical trial participation and suggests interventions such as research navigation to improve awareness, reduce barriers, and support enrollment. These findings are intended to inform future interventions aimed at promoting equitable clinical trial access in underserved rural communities.

## 2 Methodological Orientation and Theoretical Framework

This study was guided by the Integrative Behavioral Socioecological Framework (IBSF), a synthesized framework developed to support study design and implementation. IBSF was developed through an iterative theoretical synthesis process aimed at producing a unified analytic structure capable of organizing behavioral determinants across cognitive, motivational, social, and environmental domains. The development process involved three sequential phases: framework identification and selection, theoretical synthesis and construct mapping, and matrix consolidation and refinement. IBSF integrates constructs from established behavioral theories, including the Capability, Opportunity, Motivation, Behavior (COM‐B) model, the Theoretical Domains Framework (TDF), the Health Belief Model (HBM), the Theory of Planned Behavior (TPB), and the Socio‐Ecological Model (SEM), into a unified, multi‐level structure [10, 11, 12, 13, 14].

**FIGURE 1:**
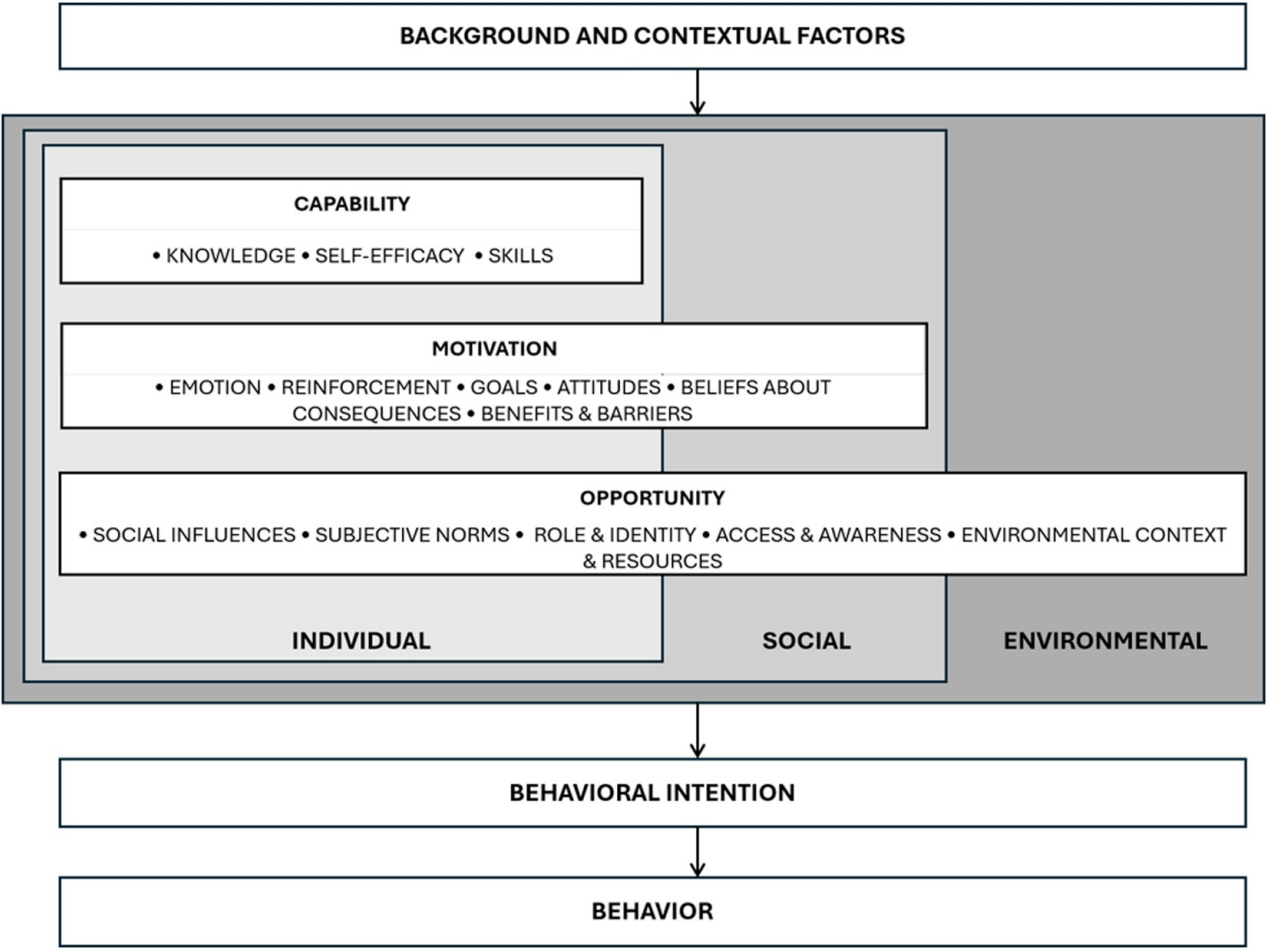
Integrated Behavioral‐Socioecological Framework (IBSF). This model integrates constructs from the Capability, Opportunity, Motivation, Behavior (COM‐B) model, the Theoretical Domains Framework (TDF), the Health Belief Model (HBM), the Theory of Planned Behavior (TPB), and the Socio‐Ecological Model (SEM) to illustrate how individual capabilities and beliefs, social influences, and environmental conditions interact to shape health‐related behaviors.

As shown in Figure 1, IBSF organizes determinants of health behavior across individual, social, and environmental levels and aligns these determinants with the domains of capability, opportunity, and motivation. The figure presents a simplified representation of the framework, reflecting a broader conceptual synthesis in which overlapping constructs from multiple theories are consolidated into higher‐order domains.

Within this structure, individual‐level factors include capability, beliefs, and motivation; social‐level factors include social influences such as norms, interpersonal relationships; support, and environmental‐level factors include access, resources, and structural conditions. Opportunity is conceptualized as spanning social and environmental domains, reflecting the interaction between contextual influences and perceived feasibility of behavior.

IBSF was applied prospectively to guide both data collection and analysis. The framework informed the development of the focus group guide to ensure coverage of determinants across levels, including individual beliefs and capabilities, social influences, and environmental constraints. During analysis, IBSF guided the development of a structured coding framework, while an inductive approach was maintained to allow for the identification of emergent themes. This combined deductive–inductive approach supported a comprehensive and theoretically grounded interpretation of participant experiences.

## 3 Methods

### 3.1 Focus Groups

This study, supported by the Florida Department of Health, Public Health Research, Biomedical Research Program (FDOH), was designed to bring cancer research closer to rural Floridians [15]. Research Patient Navigators (RPNs) were hired at FCS locations with the highest concentrations of self‐reported rural patients, as defined by Rural Urban Commuting Area Codes classification [16]. RPNs provide individualized support to patients by addressing logistical challenges, improving health literacy, and assisting with care coordination across complex healthcare systems. The FCS RPNs partnered with community centers, within their areas, to host each focus group. Data was collected via five focus groups with each session consisting of 3–8 participants.

### 3.2 Sampling

A cohort of 30 participants was established by purposively recruiting people from multiple community settings across Florida. Recruitment was conducted through the distribution of flyers in high‐traffic community areas (e.g., local libraries, religious organizations, support groups, and community centers) and via email outreach through established community networks. Potential participants were screened for eligibility based on residency in rural counties and their interest in discussing cancer clinical trials. This method of approach ensured that the sample included individuals with no prior professional ties to the research team, reducing the risk of bias in the reported perspectives. All participants were English speaking, aged > 18 years, cancer patients, survivors, caregivers or advocates, located within or near a rural community, and able and willing to provide written informed consent. No individuals who expressed interest in participation declined or withdrew from the study. No non‐participants were present during the focus group sessions.

### 3.3 Data Collection

A focus group interview guide was utilized to begin each session followed by a semi‐structured interview questionnaire. This questionnaire was tested through mock sessions conducted between members of the research team to ensure clarity and appropriateness of questions. The questionnaire focused on community awareness of CCTs, willingness to participate in clinical trials, and perceived barriers to participation. Questions were designed to capture determinants across individual, social, and environmental levels. This approach ensured the questionnaire and data collection explored how individual knowledge and beliefs, social influences, and broader environmental or structural factors shape perceptions of clinical trials and decisions regarding participation. Repeat interviews were not conducted, as the study design focused on single‐session focus group discussions.

All focus group sessions were facilitated by an RPN trained in qualitative research methods. A second member of the research team attended each session to document field notes capturing non‐verbal cues, group dynamics, and contextual observations. Each session lasted approximately 60–90 minutes and was audio‐recorded, using digital recording devices, with participants' consent. Recordings were subsequently transcribed verbatim for analysis. Transcripts were not returned to participants for comment or correction. To establish interpretive trustworthiness and confirmability without participant feedback, our team conducted regular peer debriefing sessions to audit the coding trajectory and protect against individual analyst bias. We also utilized negative case analysis by intentionally searching the dataset for disconfirming evidence or outlier perspectives regarding trial barriers. Finally, we employed rich direct quote anchoring, contextualizing the findings with verbatim quotes to create a transparent, verifiable audit trail linking raw community data to the final higher‐order themes. Data saturation was monitored throughout the data collection process. Recruitment and focus group sessions continued until no new themes or perspectives related to rural barriers to clinical trial participation emerged in subsequent discussions.

### 3.4 Data Analysis

Transcripts were analyzed using a thematic analysis approach. Two researchers independently coded the transcripts to ensure inter‐coder reliability, with discrepancies resolved through discussion and consensus‐building sessions. An inductive‐deductive analytic approach was employed. A coding framework was developed and iteratively refined, with codes organized into hierarchical categories representing emerging themes (deductive coding). As coding progressed, additional codes were incorporated to capture emerging perspectives and experiences described by participants (inductive coding). Data management and coding were conducted using Microsoft Excel and Microsoft Word.

Codes were subsequently grouped into higher‐order categories, and related categories were organized into overarching themes that reflected recurring patterns across focus groups. Throughout the analytic process, researchers reviewed transcripts iteratively to ensure that themes accurately represented participants' experiences and perspectives. IBSF was used to structure both focus group inquiry and thematic interpretation by examining how individual capabilities and beliefs, social influences, and environmental conditions interact to shape awareness of, and decisions regarding participation in CCTs (Figure 1). The reported findings were consistent with the data collected across focus groups and supported by multiple participant perspectives.

### 3.5 Ethics and Informed Consent

The study protocol and all recruitment materials were reviewed and approved by Western Copernicus Group Institutional Review Board (Protocol: Innovation_002). Prior to the commencement of each focus group, the RPN provided a detailed explanation of the study's purpose, the voluntary nature of participation, and the measures taken to ensure confidentiality. Written informed consent was obtained from all participants prior to data collection after which participants received a gift card as compensation for their time. Participants were introduced to the facilitators through a standardized script, which outlined the purpose of the study, the facilitators' roles as researchers, and the voluntary nature of participation. Participants were informed that they could withdraw from the study at any time without penalty. All transcripts were de‐identified to protect participants' anonymity, and digital data were stored on secure, password‐protected institutional servers.

### 3.6 Research Reflexivity

Focus groups were facilitated by female RPNs holding BA, BS, and MPH degrees, who engage in clinical research and community outreach. The facilitators did not have any prior relationship with the participants. To minimize power imbalances and social desirability bias, the facilitators introduced themselves as neutral researchers interested in improving community‐led interventions. The facilitators' professional background as RPNs provided a deep understanding of the structural barriers to clinical trial enrollment in Florida. Furthermore, they acknowledged their professional involvement in clinical trial navigation as a potential source of bias and engaged in reflexive practices to ensure that participant perspectives were prioritized over researcher assumptions.

## 4 Results

### 4.1 Participant Characteristics

Focus group participants were primarily female (90.0%) (Table 1). Participants self‐identified as cancer survivors (50.0%), advocates for cancer (36.7%), caregivers (33.3%), and/or currently being treated for cancer (26.7%) (Table 1). It is important to note that participants could select one or more of these identities. The county in which each focus group was held was also recorded. In the interest of maintaining confidentiality and encouraging open discussion, demographic information beyond gender, identity, and county was not collected.

**TABLE 1:** Characteristics of focus groups and participants.

Characteristic	Number of Participants	Percent (%)
Gender		
Female	27	90.0
Male	3	10.0
Identity^a^		
Cancer survivor	15	50.0
Cancer advocate	11	36.7
Caregiver	10	33.3
In treatment for cancer	8	26.7
County of focus group		
Leon	8	26.7
Marion	7	23.3
Charlotte	7	23.3
Gadsen	5	16.7
Palm beach	3	10.0

^a^ Multiple responses allowed.

### 4.2 Theme A. Perception and Lived Experience

The focus group questionnaire opened by inviting participants to reflect on their lived experiences with cancer diagnosis and care. Once themes were identified through the full data set, quotes, both positive and negative, were pulled based on their full representative nature to the theme in which they depict (Table 2). Participants shared narratives shaped by personal diagnoses, caregiving roles, and community advocacy. These stories largely reveal complex and layered experiences rather than uniformly positive or negative perceptions. Most participants described mixed experiences, often expressing deep gratitude for compassionate hospital and clinic staff while simultaneously recounting challenges within the healthcare system (A.1). Positive experiences were frequently attributed to the kindness, dedication, and emotional support provided by healthcare professionals (A.2). In contrast, negative experiences centered on presumed absence of empathy, lack of agency, treatment complexity, and the urgency associated with navigating the cancer journey (A.3). A small number of participants expressed neutral perspectives or noted limited direct experience with cancer. Overall, participant narratives reflected the emotional, logistical, and relational dimensions of cancer care, underscoring how interpersonal interactions significantly shaped perceptions of treatment experiences.

**TABLE 2:** Themes identified through focus group participant response analysis and selected representative quotes.

#	Quote
Theme A. Perception and lived experience	
A.1	Like I said 30 some years ago, clinical trials seem to be to me very secretive. And not much was told to me. And like I said, they could have talked to my husband. But as far as the healthcare and the nurses and the doctors, they were superb. This one nurse came in and she said, ‘what would you like? What would make you happy?’ and I said, ‘A shower’, and she said, 'We can do that' and I said ‘No, we can't, I've got all this stuff all over…’. She says, ‘Leave it to me.’ We went into that shower, she had bath towels, and we had water all over the place. I said, ‘They're going to come in here and raise holy…’. She says don't worry about it. And after I got well and felt like dressing up. I Went back and I saw her, and I said, ‘You have no idea what that shower did for me.’ the care that you get on these oncology floors are just absolutely wonderful. Thank you.
A.2	My experience with the cancer doctor was excellent. He treated me like a queen hoopty doo. I Was treated like royalty. Everything explained, everything taken care of.
A.3	So, I would have to say that during diagnosis, I was terrified. And I felt like everything was happening to me, and I had no control over it, that it was okay. Now we see what you have, and this is what we do, and this is what will happen next. And there were very few options presented. It was just this is the way it'll be. Maybe I didn't do enough research, but I was in shock and there was also a haste.
Theme B. Trial information dissemination	
B.1	I Would not know how to find it. It's not easy to navigate.
B.2	I Would do both. If it was me, I would do the research and also depends on the insurance. And yes, I will do a second opinion. OK, why? I Just would.
B.3	When you ask your doctor, your caregiver, your family, they're not neutral answers because everyone has a relation to it and our experience with AI is important. How do you question, and you have to complete your questioning, and I think I will find it via AI. But not at one night. It will take days. Because when you go into AI, you go deeper and deeper and deeper and then you can make your own decision about your own impression. Yeah, AI would for me be the answer.
Theme C. Enrollment impediments	
C.1	But again, that elephant in the room is always like our access you know distrust in the African American/Black community when it comes to our medical doctors. Don't feel like you get all the information.
C.2	Now, I'm going back and sharing it because I do think when you say rural and that we don't change our mindset. We let additions and generations of thought processes stay there forever. Like you have very few people that have challenged their children to go on and be all you can and come back, and you know what happened. So, it's a church thing because. Mm‐hmm. Did all of this stuff that was medicine back there for us. It's a different type of medicine, not just spiritual. But, you know, helping the rural people, the farmers, people who are just telling their children don't go into the city and trust them big city folks. We have to change all of that negative thoughts and words that say it.
C.3	But as far as me being in a clinical trial to try out different doses of the medication honestly, what for me it would be like, is it going to cause nausea, vomiting and also how far do I have to travel and how frequently? If it's really going to impinge my lifestyle and make me feel sick, then heck no.
C.4	I'd have to do a lot of research. And I don't trust 99% of the things the medical establishment says. I mean, after all, they told me coffee would kill me. And then they told me coffee is good for you. Then they said it'll give you cancer. Then they said it will cure cancer and then they said it. I'm drinking my coffee along with anything else.
C.5	Nothing. I'm going for it. I don't have a car, but I get where I need to go when it is something important.
C.6	I Would probably be concerned about location. If it's not here, where would I be and what would be the cost? Would I be responsible for it? How long would it take, and so on? There are a lot of factors I have to think about before going forward.
Theme D. Intervention strategies and implementations	
D.1	Raising awareness of clinical trials through PR, through social media, but I think directly to the patient, and I think through a town hall or something like bringing in experts. But the fact of the matter, they're only talking to other physicians. They're not talking to you, us, and I think that's part of that.
D.2	So, the numbers are one in eight in terms of breast cancer, and I think that number is significant enough to justify PSAs on TV to put out the kind of information that we've been talking about, about a support group, about clinical trials. That's what the stations are designed to do.
D.3	I Think educating cause we even talking today, we all have different ideas of what a clinical trial is. So, I think starting just with basic education, when we're in the waiting room or when we're at all of our appointments understanding what a clinical trial even is.
D.4	Bring it to us. It just is as simple as that. Make it convenient, make it closer to us, more convenient to us, for us to participate.

Patient perspectives on diagnosis, caregiving, advocacy, provider interactions, and system navigation map to the IBSF domains of individual motivation, social opportunity (interpersonal relationships), environmental opportunity (treatment and system complexity), and individual capability (experience, knowledge, and agency).

### 4.3 Theme B. Trial Information Dissemination

Following a brief overview of CCTs, participants were invited to discuss their understanding, information‐seeking behaviors, and willingness to participate. While general awareness of CCTs was present among many participants, discussions revealed varying depths of understanding. Some participants demonstrated familiarity with the concept of clinical trials, whereas others expressed uncertainty or articulated misconceptions about their purpose and safety. Some participants expressed that they were unaware of how to access information about clinical trials. (B.1). When considering participation, responses reflected cautious openness. A portion of participants expressed clear willingness to enroll, often framing participation as an opportunity to help others or access new treatments. Half of focus group participants described conditional willingness, emphasizing the need for more information, reassurance, and trust before making such a decision (B.2). Only a few participants indicated firm opposition. Physicians emerged as the most trusted and frequently cited source of information. Several participants stated they would rely exclusively on their doctor's guidance when making decisions about trial participation. Others described supplementing physician input with online research, media sources, or conversations with trusted family and friends. During the conversation, artificial intelligence (AI) as a potential informational resource was discussed and agreed to be a provider of neutral information (B.3). Overall, the discussions highlighted the significant role of trust, credibility, and accessibility in shaping how individuals seek and interpret information about CCTs.

These information dissemination thoughts span all behavioral dimensions of IBSF, where trial familiarity, uncertainty, misconceptions, and information‐seeking reflect individual capability; access and resource awareness represent individual and environmental opportunity; reliance on clinicians, family, and friends reflects social opportunity; trust, perceived outcomes, and willingness drive individual motivation.

### 4.4 Theme C. Enrollment Impediments

Participants identified multiple barriers that could discourage enrollment in clinical trials. These barriers included concerns related to trust, logistical challenges, and the potential burden associated with participation. Distrust in the healthcare system emerged as an important factor influencing attitudes toward clinical research, particularly within historically marginalized communities. (C.1). Participants also described cultural and generational influences within rural communities that may contribute to hesitancy toward healthcare institutions (C.2). Beyond trust‐related concerns, participants emphasized practical considerations when evaluating clinical trial participation. Travel requirements, potential side effects, and disruptions to daily life were commonly discussed. (C.3). Additionally, some participants expressed broader skepticism toward medical information and recommendations, reflecting uncertainty about changing scientific guidance. (C.4) Overall, participants expressed conditional willingness to enroll in a clinical trial if it was recommended and thoroughly explained by a trusted provider, supported by adequate resources including transportation assistance, and side effects caused minimal disruption to daily routines.

#### 4.4.1 Rural Barriers

When reflecting specifically on rural communities, participants again emphasized geographic distance and transportation as defining challenges. Travel to larger medical centers was described as inconvenient and, at times, prohibitive. However, some participants did not perceive rural residence alone as a primary barrier (C.5). Social considerations, including concerns about burdening family members for transportation or support, were also raised. Time constraints were noted as an additional challenge, particularly when travel required absences from work or home responsibilities. (C.6).

Participant enrollment impediment discussions concentrated across the IBSF domains of environmental opportunity (logistics like travel, time, and resources), social opportunity (family burden, cultural influences, and system distrust), individual capability (the need for clear trial explanations), and individual motivation (side effect concerns, distrust, and conditional willingness).

### 4.5 Theme D. Intervention Strategies and Implementation

Participants proposed several strategies to improve awareness and accessibility of clinical trials. A key recommendation centered on improving communication between researchers, healthcare providers, and community members. Discussions emphasized the need for outreach efforts that directly engage patients rather than focusing solely on communication within professional medical networks. (D.1). One participant suggested expanding community‐based educational opportunities (D.2). Others highlighted the potential value of broader public education campaigns to increase awareness of clinical trials and available support resources. Many also stressed the importance of education about trials within clinical settings, noting that patients often lack a clear understanding of what clinical trials involve (D.3). Finally, improving accessibility by bringing clinical trial opportunities closer to rural communities was identified as a key strategy for increasing participation. (D.4). Overall, participants suggested that improving education, trust, and accessibility could significantly enhance engagement with clinical trials.

These engagement responses align with the IBSF domains of individual capability (education and discussions), individual opportunity (information access and resource awareness), social opportunity (communication among researchers, providers, patients, and communities), environmental opportunity (rural community access), and individual motivation (trust‐building, reduced barriers, and willingness to engage).

All participant responses fell under the umbrella of these four themes, and no outlying responses were received.

## 5 Discussion

Collectively, our findings contribute new qualitative insight into how rurality, trust, and logistical constraints intersect to shape clinical trial decision‐making, offering a more nuanced understanding of the multifaceted barriers and facilitators that influence participation.

### 5.1 Individual Experiences and Trusted Sources of Information

Although distrust in the medical system and negative past experiences with healthcare frequently appear in studies on cancer care, our review found minimal literature discussing these barriers specifically in the context of clinical trials [17]. This gap is noteworthy, as many participants now view clinical trials as an extension of healthcare, a shift in perception compared with previous years [18]. While this shift is promising, it also introduces new potential barriers to participation and corresponds with our findings of patient conditional willingness to clinical trial participation.

The factors identified by our focus group participants are widely recognized in existing literature as key facilitators of trial participation [18, 19]. As participants emphasized how media platforms they use shape their awareness and perceptions of clinical research, collaborating with sponsors and researchers to leverage preferred media sources may increase trial visibility within rural communities and, by extension, enhance transparency and accountability surrounding clinical trials. Furthermore, participants reported using AI for health information, which appears unique within current literature. This likely reflects the rapid recent adoption of AI tools among the public. As more medical centers incorporate AI into recruitment and pre‐screening workflows, our results suggest an opportunity to extend these innovations to patient‐facing, trial‐specific AI tools. Such tools may deliver clear, impartial information, support decision‐making, and further reduce informational barriers to participation.

### 5.2 Structural, Logistical, and Emotional Barriers to Enrollment

Transportation and geographic challenges, as well as time and financial pressures, are well‐documented in prior research and are considered among the most substantial obstacles to participation, particularly for rural populations [3, 4, 7, 20].

These barriers illustrate how structural and logistical factors can shape participation decisions even when individuals express genuine interest in clinical trials. This underscores how environmental and social factors intersect to influence decision‐making. Within the IBSF framework guiding this study, these themes extend beyond opportunity‐related determinants alone, illustrating how structural and social barriers interact with individual understanding, trust, perceived barriers, and beliefs about clinical trial participation.

## 6 Implications

### 6.1 Clinical Trial Access and Future Research

Understanding how populations seek and receive information about clinical trials and health concerns is essential for determining effective outreach strategies. The findings from this study suggest that decisions about clinical trial participation are shaped by multiple intersecting determinants, including individual knowledge and lived experiences, trusted social relationships, and structural factors that influence access to care. The use of the IBSF in this study provided a helpful framework for understanding how these factors interact and may guide future efforts to increase clinical trial awareness and accessibility within rural communities.

To advance the goal of bringing research opportunities to rural populations and strengthening trust in the medical system, we recommend implementing mobile research units and telehealth‐based research appointments. Both strategies have demonstrated improvements in trial participation and have been met with positive patient feedback [21, 22].

A key component in bridging this gap, even with telehealth practices and mobile units in place, is the role of the RPN. Healthcare providers face their own barriers to engaging effectively with patients, including overwhelming workloads, limited time, and communication challenges. While provider training in these areas is beneficial, RPNs enter the clinical context already equipped with the skills and dedicated time needed to bridge these gaps [23]. Beyond filling healthcare gaps, RPNs build trusting relationships that are foundational for engagement in clinical research. This relational support is especially vital in rural communities, where trust, familiarity, and personalized communication heavily shape willingness to participate.

These findings underscore the importance of addressing structural, relational, and informational barriers simultaneously to build more equitable, patient‐centered clinical trial systems that effectively serve rural communities. Future research should examine how these interventions perform across diverse rural settings, identify which components are most effective in building trust, and explore tailored communication approaches that address the distinct needs of different subpopulations.

## 7 Limitations

Some limitations should be considered when interpreting these findings. First, the study population was overwhelmingly female, which may skew the identified barriers and facilitators toward perspectives more common among women. Male‐specific concerns may be underrepresented in these findings, with prior research showing that men differ in healthcare seeking behaviors, trust in healthcare systems, and perceptions of cancer risk [24]. Second, the geographic scope was limited to five rural areas in Florida. While participants across these regions shared similar experiences, their perspectives may not fully capture the diverse cultural, economic, or logistical challenges faced by rural populations in other states or other parts of Florida. While we assured data saturation was met with our small sample size, rural communities differ substantially in demographic composition, cultural norms, socioeconomic conditions, healthcare infrastructure, and transportation access. Furthermore, while healthcare‐based AI systems are promising for patient use, publicly available AI tools do not always provide accurate health information and therefore may mislead patients in their search for clinical trial information [25]. Finally, the reliance on self‐reported data from focus groups introduces the potential for recall or social desirability bias, where participants may have felt pressured by group dynamics to align with more “acceptable” viewpoints. Future research should aim for a gender‐balanced sample and explore these themes across a broader range of rural settings to validate the generalizability of our findings. Despite these potential limitations, this study provides meaningful insight into the interconnected structural, relational, and informational barriers that shape clinical trial participation for rural patients. By deepening our understanding of these factors, we can design more patient‐centered research environments that support informed decision‐making and promote broader participation in clinical trials across rural communities.

## 8 Conclusions

This study offers new insight into how rural residents understand, evaluate, and engage with cancer clinical trials, demonstrating that participation decisions arise from the interaction of trust, prior healthcare experiences, logistical constraints, and the availability of clear, actionable information.

Our findings outline how clinical trial experiences, including patient perspectives, information sharing, enrollment barriers, and engagement strategies, map across IBSF behavioral dimensions. Ultimately, successful trial participation relies on optimizing patient capability and motivation while removing environmental and social obstacles. This information also contributes several novel perspectives to the literature: the use of AI tools as emerging sources of health information, the presence of mistrust directed specifically toward clinical research (distinct from general medical distrust), and the observation that many participants now view clinical trials as part of routine healthcare, an important shift in perception. Participants also emphasized the importance of trusted messengers, the persistence of trial‐related misconceptions, and the need for information delivered in personalized, locally grounded formats rather than traditional, urban‐centered recruitment approaches.

These insights point toward practical pathways for engagement. RPN‐led outreach, enhanced physician communication, and community‐preferred media strategies may be especially effective in strengthening trust, demystifying the trial process, and addressing misconceptions around safety, cost, and local availability. Implementing structured educational programs, delivered through navigators, clinicians, or community partners, can further reinforce these efforts by providing clear, accessible information tailored to rural patients' concerns and learning preferences. Emerging technologies, such as patient‐centric AI tools, also offer promise in delivering impartial, easy‐to‐understand trial explanations to individuals who may hesitate to rely solely on clinical staff. When combined with structural supports such as mobile research units and telehealth trial appointments, these strategies can work together to reduce long‐standing barriers to participation and create a more equitable pathway into clinical research for rural communities.

Together, these findings underscore the need for rural engagement approaches that integrate trust‐building, community‐specific communication, and thoughtful interventions. Ultimately, this study suggests that achieving equitable access to oncologic care requires a patient‐centric approach that combines RPN‐led outreach with structural interventions. Future efforts should focus on integrating these navigation strategies into the standard of care to ensure that a patient's zip code does not dictate their ability to participate in life‐saving research.

## Author Contributions

Monk, B.J., Vigal, K., Patel, C., and Bentz, L. contributed to the research grant development and study concept. Cater, V. Tagurum, Y. Gonzales, G.A., and Kiplagat K.J. contributed to study design, implementation, systematic review and risk of bias assessment. Data collection was performed by Cater, V. Tagurum, Y., Gonzales, G.A., Kiplagat K.J., Pickles, H. Data analysis was conducted by Cater, V. Tagurum, Y., Gonzales, G.A., Kiplagat K.J., and Pickles, H. Wells, J.N. and Tagurum, Y. contributed to framework development. Tagurum, Y., Wells, J.N., Cater, V., Gonzales, G.A., Kiplagat, K.J. and Pickles, H. drafted the manuscript. All authors critically reviewed the manuscript and approved the final version.

## Ethics Statement

The study protocol and all recruitment materials were reviewed and approved by the Western Copernicus Group Institutional Review Board.

## Conflicts of Interest

The authors declare no conflicts of interest.

## Data Availability

The data that support the findings of this study are available from the corresponding author upon reasonable request.
